# 6 Hz Active Anticonvulsant Fluorinated N-Benzamide Enaminones and Their Inhibitory Neuronal Activity

**DOI:** 10.3390/ijerph15081784

**Published:** 2018-08-20

**Authors:** Isis J. Amaye, Thomas Heinbockel, Julia Woods, Zejun Wang, Miguel Martin-Caraballo, Patrice Jackson-Ayotunde

**Affiliations:** 1 Department of Pharmaceutical Sciences, School of Pharmacy and Health Professions, University of Maryland Eastern Shore, One Backbone Road, Princess Anne, MD 21853, USA; ijamaye@umes.edu (I.J.A.); mmartin@umes.edu (M.M.-C.); 2 Department of Anatomy, College of Medicine, Howard University, 520 W Street, NW, Washington, DC 20059, USA; theinbockel@Howard.edu (T.H.); julia.woods@bison.howard.edu (J.W.); zejunwang@hotmail.com (Z.W.)

**Keywords:** anticonvulsant, brain, calcium channel, drug discovery, epilepsy, enaminones, electrophysiology, GABA, neuron, sodium channel

## Abstract

A small library of novel fluorinated N-benzamide enaminones were synthesized and evaluated in a battery of acute preclinical seizure models. Three compounds (GSA 62, TTA 35, and WWB 67) were found to have good anticonvulsant activity in the 6-Hz ‘psychomotor’ 44-mA rodent model. The focus of this study was to elucidate the active analogs’ mode of action on seizure-related molecular targets. Electrophysiology studies were employed to evaluate the compounds’ ability to inhibit neuronal activity in central olfactory neurons, mitral cells, and sensory-like ND7/23 cells, which express an assortment of voltage and ligand-gated ion channels. We did not find any significant effects of the three compounds on action potential generation in mitral cells. The treatment of ND7/23 cells with 50 µM of GSA 62, TTA 35, and WWB 67 generated a significant reduction in the amplitude of whole-cell sodium currents. Similar treatment of ND7/23 cells with these compounds had no effect on T-type calcium currents, indicating that fluorinated N-benzamide enaminone analogs may have a selective effect on voltage-gated sodium channels, but not calcium channels.

## 1. Introduction

Epilepsy is a chronic and often progressive neurological disorder. It affects approximately three million people in the United States and 65 million worldwide [1]. About one in every 26 Americans will be diagnosed with epilepsy at some point in their lifetime [1]. Epilepsy is characterized by brief spontaneous recurrent, convulsive, and non-convulsive seizures caused by abnormal synchronous neuronal discharges in the brain [2]. Epileptic seizures are divided into two main categories: (a) generalized seizures and (b) focal seizures. Generalized seizures begin with the electrical neuronal discharge affecting the entire brain, whereas in focal seizures, the abnormal neuronal discharge is limited to one area of the brain. The management of epilepsy can be challenging due to the inherently complex nature of the disorder [3]. The goal for treatment of the epilepsies is complete seizure freedom without major drug-induced side effects. Despite the optimal use of available antiepileptic drugs (AEDs), 25%–30% of patients are considered to have refractory or drug-resistant epilepsy (DRE) [4]. According to the International League Against Epilepsy, DRE is defined as the “failure of a patient’s seizures to respond to at least two antiepileptic medications that are appropriately chosen and used for an adequate period to achieve and maintain seizure freedom” [5]. DRE is becoming more of an increasing burden in the epilepsy community with no known treatments. As a result, there is a need for the development of novel therapeutics for the management of DRE. Due to the clinical need for therapies to address DRE, our aim is to design, synthesize, and biologically evaluate novel benzamide enaminones as potential anticonvulsant agents. We elucidate the mechanism(s) of action on seizure-related molecular targets of the active analogs.

In our previous studies, we found the enaminone system to be an excellent pharmacophore for anticonvulsant active analogs [6,7,8,9,10,11,12]. Enaminones, the enamine of β-dicarbonyl compounds, are well known as versatile building blocks for the synthesis of various heterocycles [12]. The enaminone scaffold, which is the core component of all of our compounds, is a system comprised of a conjugated system with the general formula of: −NH−CH=CH−(CH_2_)_x_−C=O. The extensive research with anticonvulsant enaminone derivatives has shown that the analogs appear to act through two modes of action: (a) inhibition of the sodium channel and (b) via the GABAergic pathway [6,7].

**Figure 1:**
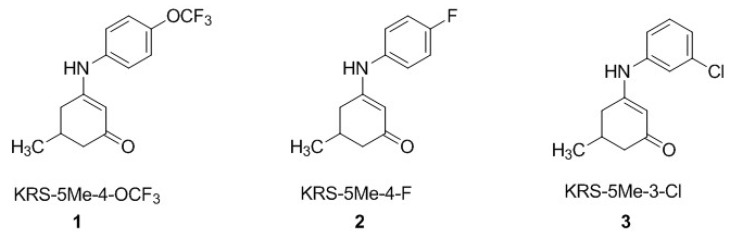
Monosubstituted aniline enaminones. The structural core of the analogs is the 5-methyl cyclic enaminone. The aromatic ring is monosubstituted at the para or meta position.

**Figure 2:**
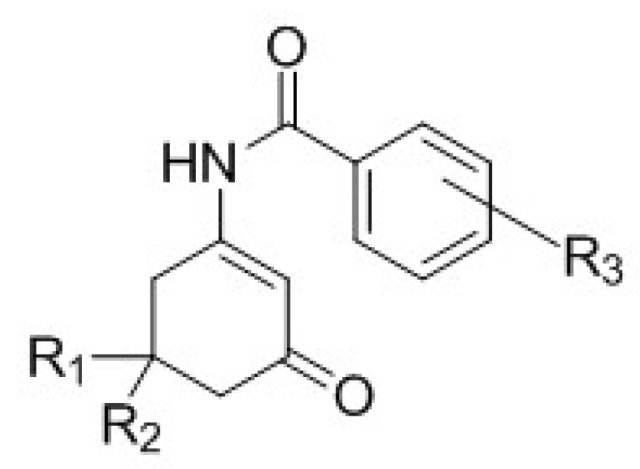
N-benzamide enaminone general structure. R_1_ = CH_3_, R_2_ = H or R_1_ = R_2_ = CH_3_; R_3_ = various aryl substitutions.

Previous work by Wang et al. [8] showed the neuronal activity of *para* and *meta*-substituted anticonvulsant aniline enaminone derivatives (Figure 1) on mitral cells in an olfactory bulb brain slice preparation using whole-cell patch-clamp recordings. Data from our electrophysiology studies revealed that the lead analog KRS−5Me−4−OCF_3_ (1) showed activity as a positive allosteric modulator of GABA at the GABA_A_ benzodiazepine receptor site [8]. Using lead-based drug design methods, we proposed that optimizing the KRS−5Me−4−OCF_3_ analog with an amide bridge functionality between the enaminone intermediate and the aromatic ring while maintaining the fluorinated group on the benzene ring (as seen in Figure 2) will allow for the analogs to retain anticonvulsant activity with better efficacy and little to no neurotoxicity. 

**Figure 3:**
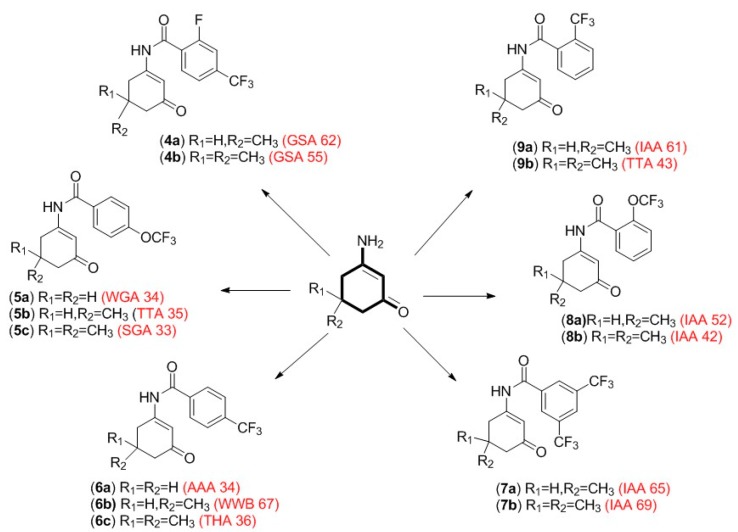
Library of fluorinated N-Benzamide enaminones. The 14 novel fluorinated N-benzamide enaminones were synthesized in moderate to good yield.

By utilizing structure–activity relationship (SAR) studies, which is a key step in the early drug discovery process for uncovering ‘hit to lead’ compounds, we have successfully synthesized a library of 14 novel fluorinated N-benzamide enaminone analogs (Figure 3). Several analogs have been screened in the acute rodent seizure models at the National Institute of Neurological Disorders and Stroke (NINDS), NIH Epilepsy Therapy Screening Program (ETSP). The animal models include: the maximal electroshock (MES) test, the 6-Hz ’psychomotor’ 44-mA test, and a separate rotarod test used to detect neurotoxicity. Each model evaluates a compound’s ability to prevent the onset of induced-seizures. The 6-Hz ‘psychomotor’ 44-mA test, in particular, correlates with DRE in humans and identifies compounds that can block a psychomotor seizure induced by long-duration (3 s) and low-frequency (6 Hz) stimulation [13,14]. To date, three lead N-benzamide enaminone compounds—GSA 62 (**4a**), TTA 35 (**5b**) and WWB 67 (**6b**)—have emerged as the first enaminone analogs to block seizures in the drug-resistant 6-Hz ’psychomotor’ 44-mA tests with limited to no observed neurotoxicity. Based on these results, we have launched molecular target studies to identify the plausible mechanism of action(s) for the active compounds. We have evaluated the inhibition of neuronal activity for the lead anticonvulsant fluorinated enaminones using whole-cell patch-clamp electrophysiology studies with a focus on seizure-related molecular targets, such as GABAergic modulation and voltage-gated sodium, and calcium channels. The results of these studies are detailed in this paper.

## 2. Materials and Methods

### 2.1. Chemistry

The N-benzamide compounds were synthesized by amination of the respective β-diketones, followed by N-acylation of the enaminone intermediates with corresponding aromatic acyl chlorides in a base-catalyzed reaction [15]. The 5-methyl and 5,5-dimethyl amination reactions were carried out with ammonium acetate as the amine source and base using anhydrous benzene as the solvent. We utilized a dean stark trap to collect the water produced in the reaction. The purified amines underwent acylation with respective mono or disubstituted acyl chlorides to generate the desired analogs. The reactions were monitored using thin layer chromatography (TLC) and gas chromatography mass spectroscopy (GCMS) chromatographic methods until reaction completion. The final reaction was quenched and extracted using dichloromethane. The combined organics were concentrated under reduce pressure to yield crude product, which was purified by column chromatography methods. All of the target analogs were obtained in quantifiable yields varying from 30%–70% yield. Proton nuclear magnetic resonance (^1^H NMR) spectra of the synthesized compounds were measured in d^6^-DMSO using TMS as an internal standard on a Bruker 400-MHz spectrometer. The purity of the compounds was assessed by TLC and GCMS. Elemental analysis for C, H and N were carried by Micro-Analysis, Inc. (Wilmington, DE, USA). The elemental analyses results were within ±0.4% of the theoretical values. Melting points were determined in open capillaries and are uncorrected. All of the chemicals and solvents were purchased from Sigma-Aldrich (St. Louis, MO, USA) and were used without further purification. 

### 2.2. Biological Testing/Anticonvulsant Activity

The in vivo evaluation of anticonvulsant activity in the MES and 6-Hz test, and the determination of neurotoxicity in the rotarod test, were performed at the University of Utah as part of the ETSP NINDS, NIH. Animal testing was performed in a manner consistent with a protocol approved by the Institutional Animal Care and Use Committee at the University of Utah (protocol 12-11011 and 15-10007, PI: Wilcox) [13,14,16,17,18]. 

### 2.3. Electrophysiology Studies—Slice Preparation

Wild-type mice (C57BL/6J, Jackson Laboratory, Bar Harbor, ME, USA) were used in agreement with Institutional Animal Care and Use Committee and NIH guidelines. Juvenile (16–25-day-old) mice were decapitated, and the main olfactory bulbs (MOBs) were dissected out and immersed in artificial cerebrospinal fluid (ACSF, see below) at 4 °C, as previously described [8]. Horizontal slices (400-µm thick) were cut parallel to the long axis using a vibratome (Vibratome Series 1000, Ted Pella Inc., Redding, CA, USA). After 30 min at 30 °C, slices were incubated in a holding bath at room temperature (22 °C) until use. For recording, a brain slice was placed in a recording chamber mounted on a microscope stage and maintained at 30 ± 0.5 °C by superfusion with oxygenated ACSF flowing at 2.5–3 mL/min.

#### 2.3.1. Electrophysiological Recording and Data Acquisition on the Slide Preparation

Visually-guided recordings were obtained from cells in the mitral cell layer with near-infrared differential interference contrast optics and a BX51WI microscope (Olympus Optical, Tokyo, Japan) equipped with a camera (C2400-07, Hamamatsu Photonics, Hamamatsu, Japan). Images were displayed on a Sony Trinitron Color Video monitor (PVM-1353MD, Sony Corp., Tokyo, Japan). Recording pipettes (5–8 MΩ) were pulled on a Flaming-Brown P-97 puller (Sutter Instrument Co., Novato, CA, USA) from 1.5 mm O.D. borosilicate glass with filament. Seal resistance was routinely >1 GΩ and liquid junction potential was 9–10 mV; reported measurements were not corrected for this potential. Data were obtained using a Multiclamp 700B amplifier (Molecular Devices, Sunnyvale, CA, USA). Signals were low-pass Bessel filtered at 2 kHz and digitized on a computer disc (Clampex 10.1, Molecular Devices, San Jose, CA, USA). Data were also collected through a Digidata 1440A Interface (Molecular Devices, San Jose, CA, USA) and digitized at 10 kHz. Holding currents were generated under control of the Multiclamp 700 B Commander. 

Membrane potentials were calculated from the steady-state membrane potential that occurred after a single action potential. To reduce the variance of spontaneous mitral cell firing rate, mitral cells with a firing rate of 1–6 Hz were used for testing cannabinoid actions. Tests for statistical significance (*p* < 0.05) were performed using a paired Student’s *t*-test, and a non-parametric Wilcoxon signed rank test for paired data of small sample sizes (~5), or one-way ANOVA followed by the Bonferroni test for multiple comparisons.

The ACSF consisted of (in mM): NaCl 124, KCl 3, CaCl_2_ 2, MgSO_4_ 1.3, glucose 10, sucrose NaHCO_3_ 26, NaH_2_PO_4_ 1.25 (pH 7.4, 300 mOsm), saturated with 95 O_2_/5% CO_2_. The standard pipette-filling solution consisted of (mM) K gluconate 125, MgCl_2_ 2, HEPES 10, Mg_2_ATP 2, Na_3_GTP 0.2, NaCl 1, EGTA 0.2.

#### 2.3.2. Chemicals and Solutions for MOB Recordings

Drugs were bath perfused at the final concentration as indicated by dissolving aliquots of stock in ACSF. The novel fluorinated N-benzamide enaminones (**4a**, **5b**–**c**, **6b**–**c**) that we tested were recently synthesized. All of the enaminones were dissolved in DMSO to make 20 mM of stock solution (final concentration of DMSO in bath <0.1%). For all of the experiments, the drugs were applied by bath perfusion. Control recordings showed that 0.1% DMSO had no detectable effects on the firing rate and membrane potential. Chemicals were supplied by Tocris (Ellisville, MO, USA) and Sigma-Aldrich (St. Louis, MO, USA). 

### 2.4. ND7/23 Cell Culture Preparation

Culture of ND7/23 cells was performed as previously described by Zhang et al. [19]. Briefly, undifferentiated ND7/23 cells were purchased from Sigma-Aldrich (St. Louis, MO, USA) and grown in DMEM-high glucose growth media supplemented with 10% fetal bovine serum, 50 U/ml penicillin and 50 µg/ml streptomycin at 37 °C in a 5% CO_2_/95% air humidified atmosphere. Cell differentiation was induced by exposing the cells to differentiation media for four to six days. The differentiation media consisted of DMEM/F12, supplemented with 0.5% fetal bovine serum, db-cAMP (1 mM), and NGF (50 ng/mL, Sigma-Aldrich, St. Louis, MO, USA).

### 2.5. Electrophysiological Recording and Data Acquisition on Cell Cultures

Whole-cell recordings of sodium and calcium currents in differentiated ND7/23 cells were performed as previously described [19]. Differentiated ND7-23 cells were visualized using a Nikon Eclipse Ti inverted microscope (Nashua, NH, USA) equipped with Hoffman optics and epifluorescence filters. Recordings were performed at room temperature (22–24 °C). Recording electrodes were made from thin-wall borosilicate glass (3–4 MΩ) and filled with a solution consisting of (in mM) CsCl (120), MgCl_2_ (2), HEPES-KOH (10), EGTA (10), ATP (1) and GTP (0.1), pH 7.4 with CsOH. Normal external saline for measurements of Ca^2+^ currents contained (in mM): tetraethylammonium chloride (TEACl, 145), CaCl_2_ (10), MgCl_2_ (1) and HEPES (10), pH 7.4 adjusted with CsOH. Normal external saline for measurements of Na^+^ currents contained (in mM): NaCl (145), KCl (5.4), MgCl_2_ (0.8), CaCl_2_ (5.4), glucose (5), and HEPES (13), pH 7.4 adjusted with NaOH. Ca^2+^ currents were generated by applying a 200-ms depolarizing step to various potentials. Na^+^ currents were generated by applying a 100-ms depolarizing step to various potentials from a holding potential of −100 mV. Voltage commands, data acquisition, and analysis were performed with a MultiClamp 700 A amplifier and Pclamp software (Axon Instruments, Foster City, CA, USA). Pipette offset, whole cell capacitance, and series resistance (usually <10 MΩ) were compensated automatically with the MultiClamp 700 B Commander. Sampling rates were between 5–10 kHz.

All of the data values are presented as mean ± SEM. Statistical analyses consisted of a Student’s unpaired *t*-test when single comparisons were made, or one-way ANOVA followed by post hoc analysis using Tukey’s honest significant difference test for unequal n for comparisons between multiple groups (STATISTICA software, Tulsa, Oklahoma). Throughout, *p* < 0.05 was regarded as significant.

## 3. Results

### 3.1. Chemistry

The synthesis of the 5-methyl and 5,5-dimethyl fluorinated N-benzamide enaminone analogs were done using a one-pot base-catalyzed acylation reaction (Figure 2) [16]. The synthetic procedure involved amination of the respective β-diketones to generate the methylated enaminone intermediates, followed by acylation with the respective substituted acyl chloride to provide target compounds **4a**–**9b**, (Figure 3) [9,15,20]. We obtained all of the final compounds in moderate to good yields (14%–63%), as shown in Table 1.

### 3.2. Pharmacology

The NINDS ETSP program selected five fluorinated N-benzamide enaminones (**4a**, **5b–c**, **6b–c**) from our drug library to test in their in vivo seizure models. The compounds underwent anticonvulsant evaluation in the initial (Identification phase) acute seizure models, MES and 6 Hz 44 mA (data shown in Table 2 and Table 3) [14,16]. The studies were done in normal adult male CF1 mice using four or more mice pre-test. Intraperitoneal (ip) administration of the test compounds were carried out as a suspension in 0.5% methylcellulose. The compounds were tested at doses ranging from 30 mg/kg to 300 mg/kg. The animals were pretreated with the test compound at time intervals from 0.25 h to 4 h. The rotarod neurologic toxicity test was completed at each dose and time point. 

The MES test is one of the gold standard preclinical seizure models for the early identification and high throughput screening of investigational antiepileptic drugs. This test, along with the subcutaneous Metrazol (scMet) test, albeit extremely effective in identifying new antiepileptic drugs that may be useful for the treatment of human generalized seizures [17], may miss potential drug candidates that could be useful in the treatment of DRE [18]. Therefore, additional “first-pass” screening assays are necessary to identify compounds that may prove effective against drug-resistant seizures. In light of this observation, test compounds that were found to be inactive in either the MES or scMet tests are screened for their ability to block psychomotor focal seizures induced by a low-frequency (6 Hz) 44-mA, long-duration (3 s) stimulus delivered through corneal electrodes in normal rodents [18]. The compounds effective in the 6-Hz model during the Identification phase become candidates for advanced screening in the Differentiation phase [13].

As shown in Table 2, the inactivity for the analogs tested in the MES mice model were consistent at all doses and pretreatment times, with the exception of GSA 62 (**4a**). Zero out of four animals were protected for WWB 67 (**6b**), THA 36 (**6c**), TTA 35 (**5b**), and SGA 33 (**5c**). GSA 62 was shown to protect the animal(s) at two time intervals and three different doses. At 0.50 h, two mice were protected at 300 mg/kg. For the pretreatment time of 2 h, 1/4 mice were protected at 30 mg/kg, 2/4 mice were protected at 100 mg/kg, and 4/4 mice were protected at 300 mg/kg.

In the 6-Hz 44-mA test model (Table 3), all five compounds showed protection at various time intervals and doses. Compound WWB 67 (**6b**) at 150 mg/kg was shown to protect 2/4, 4/4, 4/4, 2/4, and 1/4 of the animals at pretreatment times of 0.25 h, 0.50 h, 1 h, 2 h, and 4 h, respectively. THA 36 (**6c**) protected only 1/4 mice at 0.50 h at 150 mg/kg and 200 mg/kg. For analog TTA 35 (**5b**), anti-seizure protection was seen at 150 mg/kg at 0.50 h, 1 h, and 2 h. SGA 33 (**5c**) showed activity at 150 mg/kg and 300 mg/kg ranging from 1/4 to 4/4 animals protected at 0.50 h to 4 h pretreatment. Compound GSA 62 (**4a**) was tested at the same doses and time intervals as shown in the MES model (Table 2). In the 6-Hz model, GSA 62 was shown to protect 1/4 mice at 30 mg/kg (2 h), 100 mg/kg (2 h), and 300 mg/kg (0.50 h). Four out of four mice were protected at 300 mg/kg, as seen in the MES test.

### 3.3. Cell Physiological Effects of Novel Enaminones

#### 3.3.1. Mitral Cells

Recordings were obtained from mitral cells with whole-cell patch-clamp recordings in acute mouse main olfactory bulb slices. Mitral cells were identified visually by their soma location and relatively large soma size, and by their input resistance (~300 MΩ). The membrane potential of mitral cells in this study was between −50 mV and −55 mV. 

Mitral cells are principal neurons and play a crucial role in processing sensory information in the main olfactory bulb. They are the synaptic target of olfactory receptor neurons in the nasal olfactory epithelium that send their axon to the ipsilateral main olfactory bulb to form synaptic contacts with mitral cells. Mitral cells send excitatory projections to olfactory cortical areas, and receive strong feedback inhibition primarily through reciprocal dendrodendritic synapses with local interneurons [21,22]. We took advantage of the intrinsic properties of mitral cells, namely the generation of spontaneous action potentials (1–6 Hz) in slices, membrane potential, and membrane conductances to test the effect of novel enaminones on mitral cell activity and determine the possible binding target of enaminones. 

**Figure 4:**
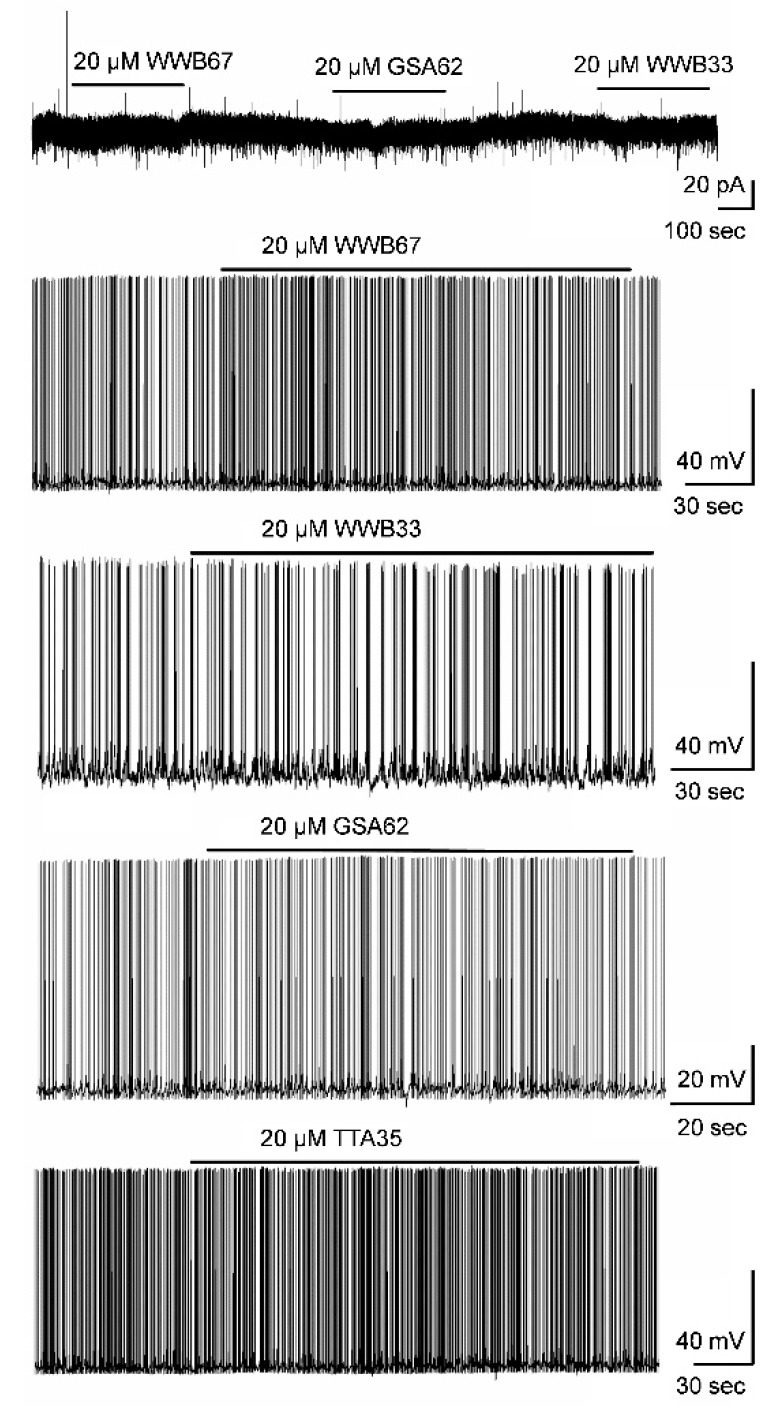
Effects of N-benzamide enaminones on membrane currents in mitral cells. Bath application of novel enaminones to test the effect on membrane currents and action potential firing in the mitral cells of the main olfactory bulb. None of the enaminones that were tested changed the firing rate or evoked distinct membrane currents. Bars above the representative recording traces indicate the application of an enaminone. WWB33 was originally termed SGA 33.

To expand our drug discovery efforts, we tested the five novel enaminones that were screened in the ETSP rodent seizure models (GSA 62 (**4a**), TTA 35 (**5b**), SGA 33 (**5c**), WWB 67 (**6b**) and THA 36 (**6c**)). We hypothesized that one or more of the five novel enaminones has a reversible inhibitory effect on mitral cells, similar to the drug effect of KRS−5ME−4−OCF that resulted in a decrease of the firing rate and a more negative membrane potential [8]. Bath application of none of the novel enaminones modulated the action potential firing rate of mitral cells or changed the membrane potential in either a depolarizing or hyperpolarizing direction (Figure 4). Likewise, the enaminones did not evoke a distinct membrane current in our recording conditions. Compared to control conditions, the recorded cells did not show measurable responses to any of the five novel enaminones (*p* > 0.05, *n* = 5).

#### 3.3.2. Cultured Cells

**Figure 5:**
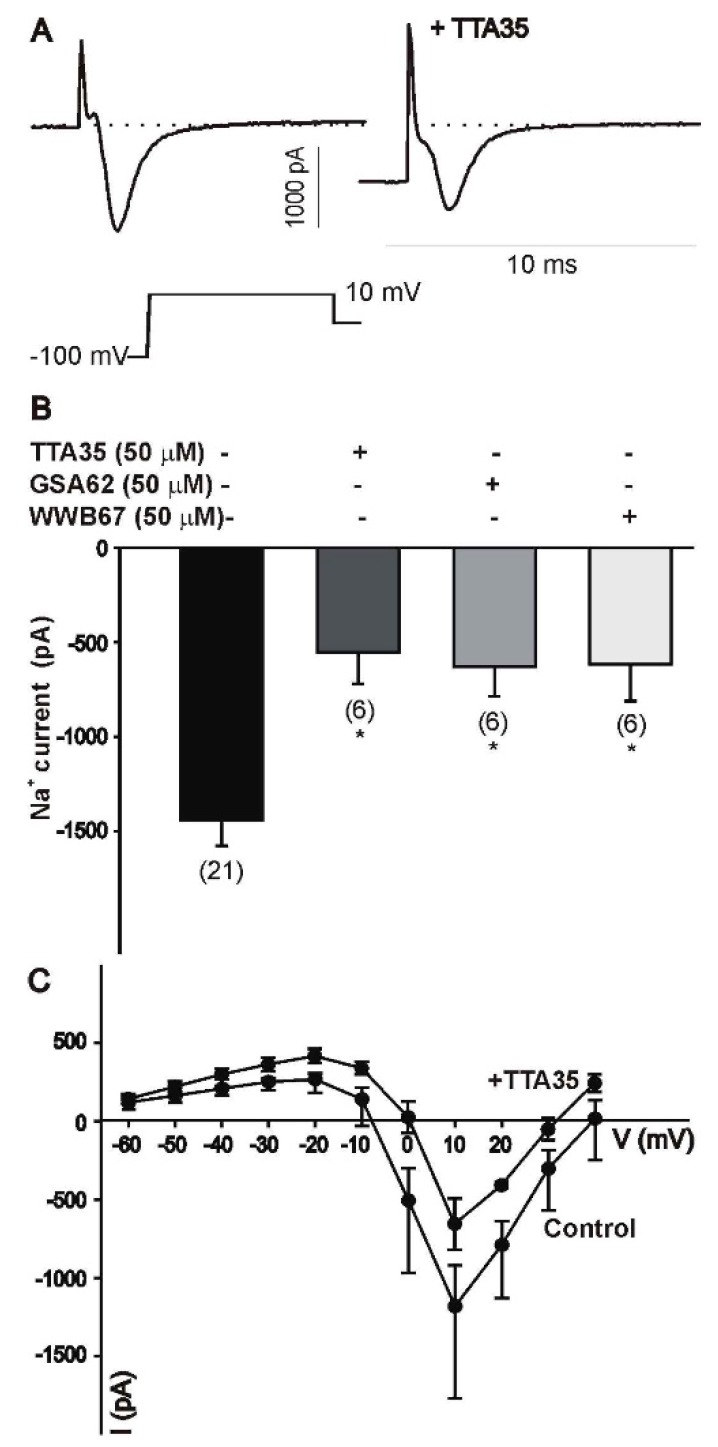
Inhibition of voltage-gated sodium channel currents. Effect of TTA 35, GSA 62, or WWB 67 on whole-cell sodium currents in ND7/23 cells. (**A**) Typical whole-cell sodium current generated in an ND7/23 cell before and after treatment with 50 µM of TTA 35. Sodium current was generated by a voltage step to +10 mV from a holding potential of −100 mV. (**B**) Treatment of ND7/23 cells with 50 µM of TTA 35, GSA 62, or WWB 67 evoked a significant reduction in the amplitude of the sodium currents generated by a voltage step to +10 mV. The number of recorded cells under each condition is presented in parenthesis. * represents *p* ≤ 0.05 vs. vehicle. (**C**) TTA 35 treatment does not alter the current–voltage relationship, as indicated by the presence of the peak current at +10 mV.

**Figure 6:**
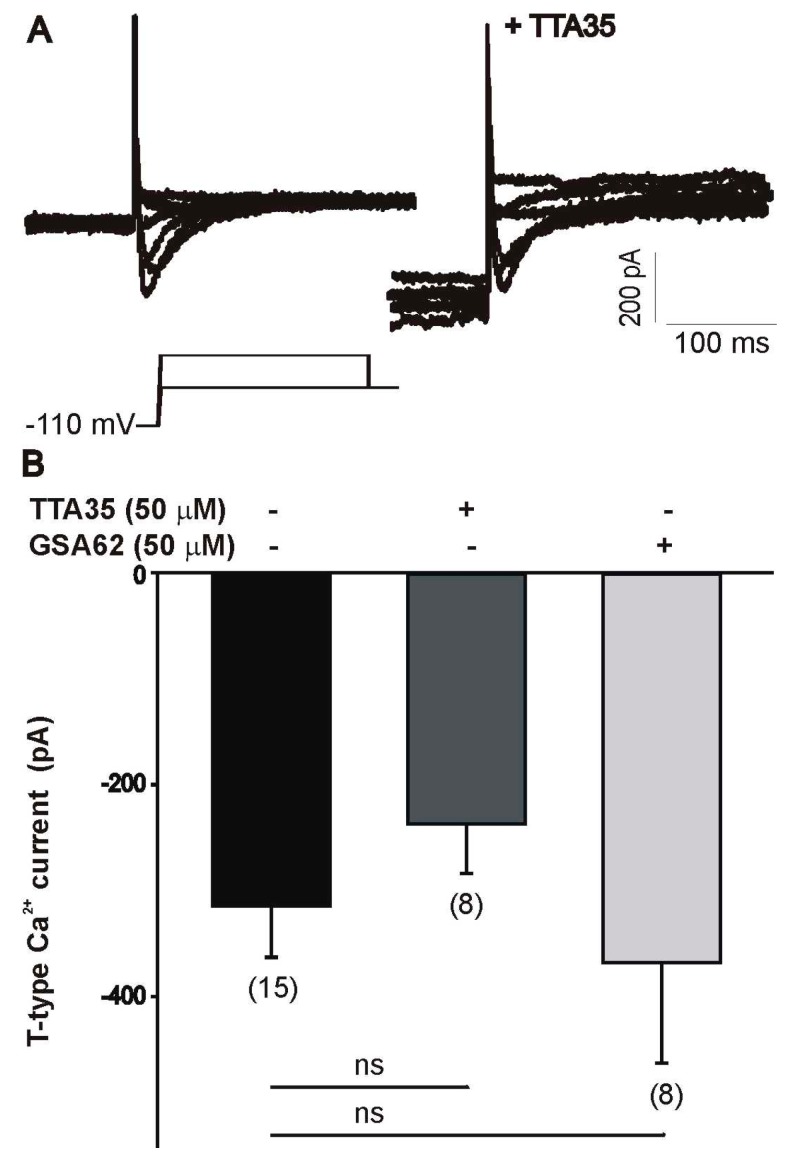
Inhibition of voltage-gated calcium channel currents. Effect of TTA 35 or GSA 62 on T-type calcium currents in ND7/23 cells. (**A**) Typical T-type calcium currents generated in an ND7/23 cell before and after treatment with 50 μM of TTA 35. T-type calcium currents were generated by a voltage step up to −20 mV from a holding potential of −110 mV. (**B**) Treatment of ND7/23 cells with 50 μM of TTA 35 or GSA 62 did not alter the amplitude of the T-type calcium currents generated by a voltage step to −20 mV. NS represents no statistically significant difference vs. vehicle.

To investigate the effect of fluorinated N-benzamide enaminone analogs on voltage-gated sodium and calcium channels, we performed whole-cell recordings using specific ionic solutions to isolate the currents of interest. As represented in Figure 5A,B, the treatment of differentiated ND7/3 cells with 50 µM of GSA 62 (**4a**), TTA 35 (**5b**), or WWB 67 (**6b**) caused a significant inhibition of sodium currents. The compounds tested did not alter the current–voltage relationship of voltage-gated sodium currents, as represented in Figure 5C using TTA 35 (**5b**) as an example. Differentiated ND7/23 cells also express a prominent T-type calcium current. However, recordings of T-type calcium channel activity revealed no changes in current amplitude following treatment with 50 µM of TTA 35 (**5b**) or GSA 62 (**4a**) (Figure 6). 

## 4. Discussion

### 4.1. Chemistry and Pharmacology

Ongoing SAR studies lead us to the design and synthesis of 14 novel mono and disubstituted fluorinated N-benzamide enaminones. The ETSP program chose five analogs of similar chemical structure with a 5-methyl or 5,5-dimethyl moiety on the cyclic enaminone structure and a para-CF_3_ or OCF_3_ at the aromatic ring. Our synthetic approach that was used to generate the target analogs were modified from previous work done by Anderson et al. [11]. The target compounds were successfully synthesized in our lab in quantifiable yields. Melting points were determined and are reported without correction. The structural identity and purity of all of the compounds were obtained using elemental analysis, GCMS, and NMR methods (data not reported). Test analogs (GSA 62 (**4a**), TTA 35 (**5b**), SGA 33 (**5c**), WWB 67 (**6b**) and THA 36 (**6c**)) showed minimal to no activity in the MES test, and moderate to excellent seizure protection in the 6-Hz 44 mA-test with no neurotoxicity (see Table 2 and Table 3). This was the first time that N-benzamide enaminones were shown to be effective against seizures in the 6-Hz 44-mA acute seizure rodent model. Our sulfonamide series (data not shown) was the first class of enaminones to show effectiveness against focal seizures in the 6-Hz 32-mA model. The latter work was recently patented (US No. 9,932,302 B1). 

Compound WWB 67 (**6b**) at a dose of 150 mg/kg was shown to have early onset activity protecting 50% of the animals at 0.25 h. The activity increases to protection of 100% of animals at two time intervals: 0.50 h and 1 h. The activity continued to extend to 2 h and 4 h. THA 36 (**6c**) was not as successful as WWB 67 (**6b**), only protecting 25% of the animals at one time interval for the 150 mg/kg and 200 mg/kg doses. TTA 35 (**5b**) and SGA 33 (**5c**) are from the same series, but similar to WWB 67 (**6b**) and THA 36 (**6c**), behaved differently in the mice. TTA 35 (**5b**) has an early onset, protecting 75% of animals at 0.50 h, whereas SGA 33 (**6c**) had the best activity at 1 h, 2 h, and 4 h, but only at 300 mg/kg, which was the highest dose tested. GSA 62 (**4a**), having two electron withdrawing substituents on the aromatic ring (2-fluoro and 4-trifluoromethyl groups) showed the best activity at 2 h, protecting the animals from the focal seizures at three different doses (30 mg/kg, 100 mg/kg, and 300 mg/kg). From these results, we concluded that the optimal dose for the active N-benzamide enaminones in the 6-Hz test is 150 mg/kg. The monosubstituted analogs (TTA 35 (**5b**) and WWB 67 (**6b**)) were shown to have better activity and potency than GSA 62 (**4a**). TTA 35 (**5b**), SGA 33 (**5c**), WWB 67 (**6b**), and THA 36 (**6c**) did not show any activity in the MES test. Only compound GSA 62 (**4a**) was shown to protect 100% of animals in the MES and 6-Hz tests at the same dose and time (300 mg/kg at 2 h), as seen in Table 3. None of the test compounds showed neurotoxicity. GSA 62, TTA 35, and WWB 67 were established as the lead compounds from the animal studies. 

### 4.2. Physiology

Our previous electrophysiological studies revealed that one enaminone compound, KRS−5Me−4−OCF_3_ (**1**), evoked significant inhibition of mitral cell activity [8]. The experiments that were aimed at understanding the cellular mechanism underlying the inhibitory effect revealed that KRS−5Me−4−OCF_3_ shifted the concentration−response curve for GABA to the left. These results indicated that KRS−5Me−4−OCF_3_ enhanced GABA affinity and acted as a positive allosteric modulator of GABA_A_ receptors. When the benzodiazepine site of the GABA receptor was blocked by an antagonist, the effect of KRS−5Me−4−OCF_3_ was blocked. This result showed that KRS−5Me−4−OCF_3_ binds at the classical benzodiazepine site to exhibit its pharmacological effect. Based on our previous finding, we hypothesized that the novel N-benzamide enaminones would show a similar inhibition of mitral cell activity. However, the lead enaminones in this study (GSA 62, TTA 35, and WWB 67) did not demonstrate robust changes in the firing rate or membrane potential of the mitral cells compared to the prominent effects of KRS−5−Me−OCF_3_ [8]. None of the tested drugs evoked significant electrophysiological responses, suggesting that they exert their activity through another mechanism of action not tested with whole-cell patch-clamp recording in an acute brain slice preparation. In the intact slice preparation, whole-cell patch-clamp recordings in current clamp mode will reveal changes in membrane potential and/or firing frequency if the underlying ionic currents are sufficiently large to change the properties of the recorded cell. Our experiments in cultured cells used recording conditions that specifically targeted changes in sodium or calcium currents. Another explanation for the lack of distinct electrophysiological responses is related to the recording conditions in a slice preparation. In cultured cells, the expression of specific ion channels might be revealed more clearly than in a slice preparation, because mitral cells are targeted by a multitude of synaptic inputs in the main olfactory bulb. The effects of these different synaptic inputs can potentially cancel each other or mask the effect on a particular ionic conductance, which makes it difficult to discern small effects on membrane potential and current. Future experiments will determine if blocking specific synaptic input, mediated for example through glutamate or GABA receptors, will reveal effects of one or more of the enaminones on central olfactory neurons. The effects on sodium channels observed in cultured ND7/23 cells might be too small to be detected in the intact circuitry of the main olfactory bulb or occur too far away from the somatic recording site. Space clamp problems, i.e., the detection of ionic currents at the distal dendrite, might make it difficult to discern ionic current when recording from the cell body of neurons. A reduction of a sodium current can be overshadowed by excitatory synaptic input that evokes firing in the recorded cell. Likewise, an allosteric modulation of GABA receptors can potentially results in disinhibitory circuit effects that evoke an increase in the firing of mitral cells. Given these experimental constraints, our results in cultured cells and mitral cells in an acute slice preparation are complementary, and will guide our future experiments.

The present results suggest that the lead compounds GSA 62 (**4a**), TTA 35 (**5b**), or WWB 67 (**6b**) have an inhibitory effect on voltage-gated sodium channels, as revealed by whole cell recordings in differentiated ND7/23 cells. However, these compounds had no effect on T-type calcium currents. These findings suggest that fluorinated N-benzamide enaminone may specifically target the conductance of voltage-gated sodium channels. Future work will investigate the mechanism of inhibition of these compounds on voltage-gated sodium channels. The ability of these compounds to target voltage-gated sodium channels may be relevant for the treatment of epilepsy generated by increased neuronal excitability. 

## 5. Conclusions

In vivo data for the mono and disubstituted N-benzamide analogs that were previously synthesized in our lab showed protection in the MES and 6-Hz 44-mA seizure rodent models with no neurotoxicity. Interestingly, we identified three lead analogs that showed protection in the rodent drug-resistant model, which is a new result that potentially has clinical implications. We were able to conclude from the electrophysiology studies that one probable mode of action for the lead analogs is the inhibition of the voltage-gated sodium channel. Other seizure-related molecular targets require further studies. The next steps are (1) to further explore the inhibitory sodium channel effects of the anticonvulsant analogs by conducting concentration dependent studies in vitro, and (2) to determine the inhibitory mechanism of these compounds on voltage-gated sodium channels, and whether they may target different subunits of the channels selectively.

## Figures and Tables

**Table 1:**
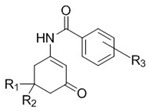
Chemical properties of fluorinated n-benzamide enaminones.

Compound	R_1_	R_2_	R_3_	Yield (%)	MP °C
**4a**	H	CH_3_	2−F, 4−CF_3_	46	170–172
**4b**	CH_3_	CH_3_	2−F, 4−CF_3_	50	177–179
**5a**	H	H	4−OCF_3_	54	157–158
**5b**	H	CH_3_	4−OCF_3_	32	186–187
**5c**	CH_3_	CH_3_	4−OCF_3_	43	170–171
**6a**	H	H	4−CF_3_	63	197–198
**6b**	H	CH_3_	4−CF_3_	48	201–203
**6c**	CH_3_	CH_3_	4−CF_3_	56	197–200
**7a**	H	CH_3_	3,5−CF_3_	34	186–188
**7b**	CH_3_	CH_3_	3,5−CF_3_	14	149–151
**8a**	H	CH_3_	2−OCF_3_	34	142–143
**8b**	CH_3_	CH_3_	2−OCF_3_	42	153–155
**9a**	H	CH_3_	2−CF_3_	21	180–181
**9b**	CH_3_	CH_3_	2−CF_3_	28	195–197

Yield percentages and melting point values obtained for synthesized analogs **4a**–**9b**.

**Table 2:**
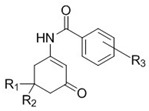
Anticonvulsant activity: maximal electroshock (MES) test in mice.

Compound	R_1_	R_2_	R_3_	Dose (mg/kg)	Pretreatment Times (h) ^a^					Tox ^b^
					0.25	0.50	1	2	4	
WWB 67 (**6b**)	CH_3_	H	4−CF_3_	100	0/4	0/4	0/4	0/4	0/4	0/4
				150	0/4	0/4	0/4	0/4	0/4	0/4
				200	0/4	0/4	0/4	0/4	0/4	0/4
THA 36 (**6c**)	CH_3_	CH_3_	4−CF_3_	150	0/4	0/4	0/4	0/4	0/4	0/4
				200	0/4	0/4	0/4	0/4	0/4	0/4
TTA 35 (**5b**)	CH_3_	H	4−OCF_3_	150	0/4	0/4	0/4	0/4	0/4	0/4
				300	0/4	0/4	0/4	0/4	0/4	0/4
SGA 33 (**5c**)	CH_3_	CH_3_	4−OCF_3_	150	0/4	0/4	0/4	0/4	0/4	0/4
				300	0/4	0/4	0/4	0/4	0/4	0/4
GSA 62 (**4a**)	CH_3_	H	2−F, 4−CF_3_	30	0/4	0/4	0/4	**1/4**	0/4	0/4
				100	0/4	0/4	0/4	**2/4**	0/4	0/4
				300	0/4	**2/4**	0/4	**4/4**	0/4	0/4

^a^ Ratios where at least one animal was protected have been highlighted in bold for easier data interpretation. Data indicate the number of mice protected/number of mice tested. Pretreatment times of the test analogs vary from as early as 15 min up to 4 h. This is Epilepsy Therapy Screening Program (ETSP) protocol to identify compounds with an early onset of action as well as a long duration of action. ^b^ Rotarod neurologic toxicity test (Tox).

**Table 3:**
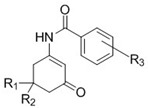
Anticonvulsant activity: 6-Hz 44-MA test in mice.

Compound	R_1_	R_2_	R_3_	Dose (mg/kg)	Pretreatment Times (h) ^a^					Tox ^b^
					0.25	0.50	1	2	4	
WWB 67 (**6b**)	CH_3_	H	4−CF_3_	100	0/4	0/4	0/4	0/4	0/4	0/4
				150	**2/4**	**4/4**	**4/4**	**2/4**	**1/4**	0/4
				200	0/4	**3/8**	**1/4**	**1/4**	0/4	0/4
THA 36 (**6c**)	CH_3_	CH_3_	4−CF_3_	150	0/4	**1/4**	0/4	0/4	0/4	0/4
				200	0/4	**1/4**	0/4	0/4	0/4	0/4
TTA 35 (**5b**)	CH_3_	H	4−OCF_3_	150	0/4	**3/4**	**1/4**	**1/4**	0/4	0/4
				300	0/4	0/4	0/4	0/4	0/4	0/4
SGA 33 (**5c**)	CH_3_	CH_3_	4−OCF_3_	150	0/4	**1/4**	**1/4**	**3/4**	0/4	0/4
				300	0/4	**1/4**	**4/4**	**3/4**	**2/4**	0/4
GSA 62 (**4a**)	CH_3_	H	2−F, 4−CF_3_	30	0/4	0/4	0/4	**1/4**	0/4	0/4
				100	0/4	0/4	0/4	**1/4**	0/4	0/4
				300	0/4	**1/4**	0/4	**4/4**	0/4	0/4

^a^ Ratios where at least one animal was protected have been highlighted in bold for easier data interpretation. Data indicate the number of mice protected/number of mice tested. Pretreatment times of the test analogs vary from as early as 15 min up to 4 h. This is ETSP protocol to identify compounds with early onset of action as well as long duration of action. ^b^ Rotarod neurologic toxicity test (Tox).

## References

[1] Citizens United for Research Epilepsy What is Epilepsy?. http://www.cureepilepsy.org/aboutepilepsy/facts.asp.

[2] Salome C., Salome-Grosjean E., Park K., Marieux P., Swendiman E.D., Stables J., Kohn H. (2010). Synthesis and anticonvulsant activities of (R)-*N*-(4′-substituted) benzyl 2-acetamido-3-methoxypropionamides. J. Med. Chem..

[3] French J.A., Staley B.A. (2012). AED treatment through different ages: As our brains change, should our drug choices also?. Epilepsy Curr..

[4] Laxer K.D., Trinka E., Hirsh L.J., Cendes F., Langfitt J., Delanty N., Resnick T., Benbadis S.R. (2014). The consequences of refractory epilepsy and its treatment. Epilepsy Behav..

[5] Choi H., Hayat M.J., Zhang R., Hirsch L.J., Bazil C.W., Mendiratta A., Kato K., Javed A., Legge A.W., Buchsbaum R. (2016). Drug-resistant epilepsy in adults: Outcome trajectories after failure of two medications. Epilepsia.

[6] Alexander S.M., Harkless J., Butcher J.R., Scott K.R., Jackson-Ayotunde L.P. (2013). Enaminones 11. An examination of some ethyl ester enaminone derivatives as anticonvulsant agents. Bioorg. Med. Chem..

[7] Heinbockel T., Wang Z., Jackson-Ayotunde P.L. (2014). Allosteric modulation of GABA_A_ receptors by an anilino enaminone in an olfactory center of the mouse brain. Pharmaceuticals.

[8] Wang Z., Sun L., Jackson P.L., Scott R.K., Heinbockel T. (2011). A substituted anilino enaminone acts as a novel positive allosteric modulator of GABA_A_ receptors in the mouse brain. J. Pharmacol. Exp. Ther..

[9] Jackson P.L., Hanson C.D., Farrell A.K., Raymond B.J., Stables J.P., Eddington N.D., Scott K.R. (2012). Enaminones 12. An explanation of anticonvulsant activity and toxicity per Linus Pauling’s clathrate hypothesis. Eur. J. Med. Chem..

[10] Anderson A.J., Nicholson J.M., Bakare O., Butcher R.J., Wilson T.L., Scott K.R. (2006). Enaminones 9. Further studies on the anticonvulsant activity and potential type IV phosphodiesterase inhibitory activity of substituted vinylic benzamides. Bioorg. Med. Chem..

[11] Jackson P.L., Scott K.R., Southerland W.M., Fang Y. (2009). Enaminones 8. CoMFA and CoMSIA studies on some anticonvulsant enaminones. Bioorg. Med. Chem..

[12] Edafiogho I.O., Kombian S.B., Anathalaskshmi K.V.V., Salama N.N., Eddington N.D., Wilson T.L., Alexander M.S., Jackson P.L., Hanson C.D., Scott K.R. (2007). Enaminones: Exploring additional therapeutic activities. J. Pharm. Sci..

[13] Kehne J.H., Klein B.D., Raeissi S., Sharma S. (2017). The National Institute of Neurological Disorders and Stroke (NINDS) Epilepsy Therapy Screening Program (ETSP). Neurochem. Res..

[14] National Institute of Neurological Disorders and Stroke The 6 Hz “Psychomotor” Seizure Test. https://panache.ninds.nih.gov/m_6hztest.aspx.

[15] Anderson A.J., Nicholson J.M., Bakare O., Butcher R.J., Scott K.R. (2004). A base-catalyzed solution-phase parallel synthesis of substituted vinylic benzamide from 3-amino-2-cyclohexanones. J. Comb. Chem..

[16] Stables J.P., Kupferberg H.J., Avanzini G., Tanganelli P., Avoli M. (1997). The NIH Anticonvulsant Drug Development (ADD) Program: Preclinical anticonvulsant screening. Molecular and Cellular Targets for Anti-Epileptic Drugs.

[17] White H.S., Bender A.S., Swinyard E.A. (1988). Effect of the selective *N*-methyl-d-aspartate receptor agonist 3-(2-carboxypiperazin-4-yl) propyl-1-phosphonic acid on [3H] flunitrazepam binding. Eur. J. Pharmacol..

[18] Barton M.E., Klein B.D., Wolf H.H., White H.S. (2001). Pharmacological characterization of the 6 Hz psychomotor seizure model of partial epilepsy. Epilepsy Res..

[19] Zhang Q.J., Hsia S.C., Martin-Caraballo M. (2017). Regulation of T-type Ca^2+^ channel expression by herpes simplex virus-1 infection in sensory-like ND7 cells. J. Neurovirol..

[20] Friary R.J., Gilligan J.M., Szajewski R.P., Falci K.J., Franck R.W. (1973). Heterocyclic syntheses via the intramolecular acylation of enamines derived from amino acids. J. Org. Chem..

[21] Ennis M., Hamilton K.A., Hayar A., Lajtha A., Johnson D.A. (2007). Neurochemistry of the main olfactory system. Handbook of Neurochemistry and Molecular Neurobiology.

[22] Shepherd G.W., Chen W.R., Greer C.A., Shepherd G.M. (2004). Olfactory bulb. The Synaptic Organization of the Brain.

